# C3d Elicits Neutrophil Degranulation and Decreases Endothelial Cell Migration, with Implications for Patients with Alpha-1 Antitrypsin Deficiency

**DOI:** 10.3390/biomedicines9121925

**Published:** 2021-12-16

**Authors:** Laura T. Fee, Debananda Gogoi, Michael E. O’Brien, Emer McHugh, Michelle Casey, Ciara Gough, Mark Murphy, Ann M. Hopkins, Tomás P. Carroll, Noel G. McElvaney, Emer P. Reeves

**Affiliations:** 1 Alpha-1 Foundation Ireland, Royal College of Surgeons in Ireland, Beaumont Hospital, D02 YN77 Dublin, Ireland; laurafee@rcsi.ie (L.T.F.); tcarroll@rcsi.ie (T.P.C.); 2 Irish Centre for Genetic Lung Disease, Department of Medicine, Royal College of Surgeons in Ireland, Beaumont Hospital, D02 YN77 Dublin, Ireland; debanandagogoi@rcsi.ie (D.G.); emmetobrien@rcsi.ie (M.E.O.); emermchugh@rcsi.ie (E.M.); michellecasey@rcsi.ie (M.C.); ciaragough@rcsi.ie (C.G.); MMurphy@rcsi.ie (M.M.); gmcelvaney@rcsi.ie (N.G.M.); 3 Department of Surgery, Royal College of Surgeons in Ireland, Beaumont Hospital, D02 YN77 Dublin, Ireland; annhopkins@rcsi.ie

**Keywords:** neutrophils, alpha-1 antitrypsin, alpha-1 antitrypsin deficiency, complement component 3d, degranulation, neutrophil elastase, myeloperoxidase, lactoferrin

## Abstract

Alpha-1 antitrypsin (AAT) deficiency (AATD) is characterized by increased risk for emphysema, chronic obstructive pulmonary disease (COPD), vasculitis, and wound-healing impairment. Neutrophils play a central role in the pathogenesis of AATD. Dysregulated complement activation in AATD results in increased plasma levels of C3d. The current study investigated the impact of C3d on circulating neutrophils. Blood was collected from AATD (*n* = 88) or non-AATD COPD patients (*n* = 10) and healthy controls (HC) (*n* = 40). Neutrophils were challenged with C3d, and degranulation was assessed by Western blotting, ELISA, or fluorescence resonance energy transfer (FRET) substrate assays. Ex vivo, C3d levels were increased in plasma (*p* < 0.0001) and on neutrophil plasma membranes (*p* = 0.038) in AATD compared to HC. C3d binding to CR3 receptors triggered primary (*p* = 0.01), secondary (*p* = 0.004), and tertiary (*p* = 0.018) granule release and increased CXCL8 secretion (*p* = 0.02). Ex vivo plasma levels of bactericidal-permeability-increasing-protein (*p* = 0.02), myeloperoxidase (*p* < 0.0001), and lactoferrin (*p* < 0.0001) were significantly increased in AATD patients. In endothelial cell scratch wound assays, C3d significantly decreased cell migration (*p* < 0.0001), an effect potentiated by neutrophil degranulated proteins (*p* < 0.0001). In summary, AATD patients had increased C3d in plasma and on neutrophil membranes and, together with neutrophil-released granule enzymes, reduced endothelial cell migration and wound healing, with potential implications for AATD-related vasculitis.

## 1. Introduction

Alpha-1 antitrypsin (AAT) is an abundant plasma protein that plays a key role in the innate immune response. The majority of circulating AAT is produced by hepatocytes and is generated as a single polypeptide chain that is post-translationally modified by glycosylation through the addition of *N*–glycosidically linked oligosaccharides [1,2]. AAT deficiency (AATD) is an autosomal codominant disorder resulting in an increased risk for the development of emphysema, panacinar in pathology, often by the third or fourth decade. AATD is caused by mutation of the *SERPINA1* gene [3]. The most common mutations known to cause AATD are the Z (Glu342Lys) and S (Glu264Val) mutations. However, there are over 100 disease-causing mutations reported to date [4,5,6]. The Z mutation occurs in approximately 95% of individuals with severe AATD [7]. The codominant expression of M/S/Z SERPINA1 gene alleles modulates the plasma levels of AAT. The normal M and S alleles are related to approximately 60% of normal AAT plasma concentrations, while the severely deficient Z correspond to approximately 15% of normal AAT levels [8]. Moreover, AAT protein levels have been reported to change, not only according to the SERPINA1 genotype but also in relation to epigenetic (DNA methylation) modifications of the gene promoter [9]. Frequent acute pulmonary exacerbations in AATD are typified by inflammation and protease burden in the airways [10], leading to the gradual decline in gas transfer in affected individuals [11].

The primary role of the AAT protein is to function as a serine protease inhibitor, as AAT has been shown to inhibit a range of proteases derived from degranulating neutrophils, including neutrophil elastase (NE) [12]. New insights into the diverse functions of AAT have, however, challenged this solitary role. AAT is now recognized as possessing important anti-inflammatory roles in key inflammatory processes, independent of its anti-protease activity, affecting inflammatory molecules such as leukotriene B_4_ [13], tumor necrosis factor-alpha (TNF-α) [14], C-X-C motif chemokine ligand 8 (CXCL8) [15], interferon-γ [16] and interleukin-1β [17]. The anti-inflammatory capacity of AAT is further determined by glycan residues, as glycosylation of AAT is altered acutely. In this regard, during the resolving phase of community-acquired pneumonia, AAT was shown to possess additional sialic acid residues that can bind increased levels of CXCL8, thereby inhibiting neutrophil chemotaxis, with a positive impact on the resolution of pneumonia [18]. 

Recently we established that a significant proportion of glycosylated AAT is bound to other abundant plasma proteins in the circulation. We identified an interaction between AAT and complement component C3 and demonstrated that a by-product of C3 cleavage, C3d, is present at increased levels in the plasma of AATD individuals [19]. Our findings indicated that deficiency in AAT results in a diminished capacity to inhibit the proteolytic processing of C3 to C3d, suggesting a potential role for complement activation in the pathogenesis of AATD-related disease [19]. In support of this concept, in ZZ-AATD individuals, higher levels of C3d were shown to correlate with worsening radiographic evidence of emphysema and a decline in lung function as assessed by forced expiratory volume in one second (FEV1). While deposition of C3d locally has been reported in lung allografts following transplantation [20] and synovial fluid samples from patients with rheumatoid arthritis [21], the role of C3d in AATD is unknown. Indeed, increased C3d may be of great importance as AATD is associated with a variety of inflammatory conditions, the best-described being ANCA-associated vasculitis and, in particular, granulomatosis with polyangiitis (GPA) [22,23]. Moreover, AATD has been proposed as a possible cause of wound healing disturbances, evident by histological patterns of necrotizing panniculitis [24]. In corroboration, evidence exists to support the use of AAT augmentation therapy in the treatment of both GPA vasculitis [25] and panniculitis [26]. As CR3 (CD11b/CD18) can bind the C3d fragment of C3 [27], the aim of this study was to investigate the consequence of C3d signaling via CR3 on neutrophils. The purpose of this study was to explore the ability of C3d to induce neutrophil degranulation and to impact endothelial cell wound repair, thereby providing a potential mechanism for the described wound healing disturbances in patients with AATD. 

## 2. Materials and Methods

### 2.1. Chemicals and Reagents

All chemicals and reagents were of the highest purity available and were purchased from Sigma-Aldrich, Dublin, Ireland unless specified otherwise.

### 2.2. Study PoPulation

Ethical approval from Beaumont Hospital Institutional Review Board was acquired (reference 13/92) and written informed consent was obtained from all study participants. The inclusion criteria for patient participants included: (1) AATD confirmed by isoelectric focusing; (2) COPD with MM-AAT phenotype; (3) aged ≥18 years old, and (4) patients capable of giving informed consent. For healthy controls (HC) the inclusion criteria included: (1) aged ≥18 years; (2) normal AAT-MM phenotype and AAT level, and (3), no respiratory illness. The exclusion criteria for all participants included: (1) aged <18 years; (2) unable to give consent; (3) pregnant or breast-feeding women, and (4), active tobacco smoking. AAT phenotyping was carried out by isoelectric focusing using the Hydragel 18 A1AT isoelectricfocusing^®^ kit on the Sebia Hydrasys^®^ System. HC (AAT sufficient) individuals (*n* = 40) were defined as having an MM phenotype with plasma AAT levels within the normal range (1.1–1.8 g/L) (Table 1). All were non-smokers and without a history of pulmonary disease. All AATD patients (*n* = 88) were recruited from the National AATD clinic in Beaumont Hospital and were confirmed as having a ZZ or MZ phenotype. AATD individuals were stable with no exacerbations at the time of recruitment. AAT sufficient COPD patients (*n* = 10) were recruited from the general respiratory clinic in Beaumont Hospital and confirmed as having an MM phenotype with plasma AAT levels within the normal range (Table 1). 

### 2.3. Isolation of Plasma and Neutrophils from Whole Blood

Blood samples were collected in Sarstedt-Monovette^®^ tubes containing lithium heparin and centrifuged at 350× *g* for 5 min at room temperature. Plasma was aliquoted for immediate use or directly stored at −80 °C. Neutrophils were isolated from whole blood samples by dextran sedimentation and Lymphoprep (Axis-Shield PoC, Fischer Scientific, Waltham, MA, USA) centrifugation as previously described [28]. Purified cells were resuspended in phosphate-buffered saline (PBS) (pH 7.4) containing 5 mM glucose and used immediately. The purity of isolated neutrophils was validated by flow cytometry analysis with a monoclonal antibody against CD16b [14,29]. Neutrophil viability was assessed by trypan blue exclusion assay. Results confirmed the viability of neutrophils greater than 98%, and the purity of isolated neutrophils was greater than 96%. 

### 2.4. Sodium Dodecyl Sulphate–Polyacrylamide Gel Electrophoresis (SDS-PAGE) and Western Blot Analysis

SDS–PAGE analyses of samples were carried out under denaturing or non-reducing conditions. Samples were electrophoresed with 12.5% (*w/v*) NuPAGE gels. After electrophoresis, gels were stained by Coomassie Blue R250 or were silver-stained for visualization of proteins, or alternatively, proteins were transferred onto 0.2 μm nitrocellulose or PVDF membrane by Western blotting with a semidry blotter for 1 h at 100 mA. The efficient transfer was verified by Ponceau S staining of membranes followed by blocking of membranes in PBS-Tween (0.05% *v/v*) containing 3% (*w/v*) nonfat dried milk and 1% (*w/v*) bovine serum albumin (BSA). 

The following primary antibodies were used for probing immuno-blots: rabbit anti-C3 (Novus Biologicals, NBP1-32080, 1 µg/mL); mouse monoclonal BPI (Santa Cruz Biotechnology, Dallas, TX, USA, H-10, 0.2 µg/mL); goat polyclonal MMP-9 (R&D Systems, Minneapolis, MI, USA, AB911, 1 µg/mL, 1:1000); rabbit polyclonal hCAP-18 (Novus Biologicals, NBP1-76864, 1 µg/mL), or mouse monoclonal β-actin (Santa Cruz Biotechnology, sc-47778, 0.1 µg/mL). Relevant secondary antibodies were all horseradish peroxidase (HRP) linked anti-goat, anti-rabbit or anti-mouse (Cell Signaling Technology, Danvers, MA, USA). Immunoreactive bands were visualized with chemiluminescent HRP substrate (Millipore). Images and densitometry were obtained on the Syngene G: BOX Chemi XL gel documentation system.

### 2.5. C3d Analysis

Production of C3d by NE cleavage of total C3 was evaluated by adding 338 nM NE (Elastin Products Company, Inc., Owensville, MO, USA) to 1% (*v/v*) HC or ZZ-AATD fresh plasma and incubated for 1 h at 37 °C. Samples were analyzed on 12.5% (*w/v*) SDS-PAGE gels under non-reducing conditions, and gels were either silver stained or Western blotted for C3d using a mouse anti-C3d antibody (Santa Cruz Biotechnology, Sc-58928). Purified C3d was included as a positive control (Merck Chemicals, Burlington, MA, USA). 

Levels of neutrophil plasma membrane C3d and cognate receptor CR3 were evaluated by flow cytometry. For this, isolated neutrophils (2 × 10^6^/mL) were fixed in 4% (*w/v*) paraformaldehyde for 10 min. Cells were then washed in PBS and blocked for 30 min with 1% (*w/v*) BSA in the presence of protease inhibitors to maintain plasma membrane receptor integrity (20 µg/mL phenylmethane sulfonylfluoride (PMSF), 10 µg/mL Na-tosyl-L-lysine chloromethyl ketone hydrochloride (TLCK), 10 µg/mL pepstatin A, 200 µg/mL Pefabloc, and 10 µg/mL leupeptinhemisulfate). After 1 h of incubation with primary antibodies, samples were washed and incubated for 30 min with relevant secondary FITC-labeled antibodies. Primary antibodies included 1 μg/mL mouse monoclonal anti-CD11b (CR3) (Novus Biologicals, Littleton, CO, USA) or mouse monoclonal anti–C3d (Abcam, Cambridge, UK). FITC-labeled mouse IgG kappa binding protein (m-IgGκ BP) (Santa Cruz Biotechnology) or FITC goat anti-mouse IgG (Santa Cruz Biotechnology) were used as secondary FITC-labeled antibodies (Santa Cruz Biotechnology).

In CR3 blocking experiments, isolated neutrophils were incubated with or without the CR3 blocker, simvastatin (Fisher Scientific, Waltham, MA, USA) (1 mM), for 30 min [27,30]. Subsequently, C3d (5 µg) (Complement Technologies, Tyler, TX, USA) binding was evaluated using C3d mouse monoclonal IgG1 antibody (Abcam) followed by FITC-labeled mouse. Subsequently, C3d (5 µg) binding was evaluated using C3d mouse monoclonal IgG1 antibody (Santa Cruz Biotechnology) followed by FITC-labeled mouse IgG kappa binding protein (m-IgGκ BP) (Santa Cruz Biotechnology). Controls for this experiment employed cells alone or cells incubated with mouse monoclonal IgG1 antibody and/or FITC labeled m-IgGκ BP. Cells were then washed, and fluorescence was measured by a BD FACSCalibur™ (Becton Dickinson, Franklin Lakes, NJ, USA). A total of 10,000 events per reaction were quantified. Analysis of fluorescence was carried out using FlowJo software, and data was represented as median fluorescent intensity (MFI).

### 2.6. Degranulation Assays

To assess degranulation of tertiary, secondary, and primary granules of circulating neutrophils (1 × 10^7^ cells/mL), cells isolated from HC individuals were challenged with increasing concentrations of C3d (0–40 µg/mL) for 10 min at 37 °C. Extracellular supernatants were then analyzed for degranulated protein as previously described [13,31]. MMP-9, hCAP-18, and BPI, markers of tertiary, secondary, and primary granule release, respectively, were detected by Western blotting. In a subset of reactions, TNF-α (5 ng/1 × 10^7^) was included as a positive control as it has previously been reported to cause secondary and tertiary granule release, but not primary granule degranulation [13,31].

The activity of neutrophil membrane-bound NE was measured by fluorescence resonance energy transfer (FRET). An NE-specific FRET substrate was used (Abz-Ala-Pro-Glu-Glu-Ile-Met-Arg-Arg-Gln-EDDnp, 3230-v, Peptide Institute, Inc., Osaka, Japan) [32]. Fluorescence was recorded at excitation 320 nm and emission 420 nm for 2–40 min on a Spectramax M3 (Molecular Devices, San Jose, CA, USA).

Ex vivo analysis of MPO (Cambridge Bioscience), lactoferrin (Cambridge Bioscience), and BPI (Hycult Biotech, Wayne, PA, USA) was measured in plasma using relevant quantitative sandwich enzyme-linked immunosorbent assays (ELISA), as per the manufacturer’s instructions.

### 2.7. CXCL8 Secretion Analysis

The HL-60 neutrophil-like cell line (American Type Culture Collection, Manassas, VA, USA; CCL-240) was cultured in complete RPMI media containing 10% (*v/v*) heat-inactivated fetal calf serum (FCS) (Cambridge Bioscience, Cambridge, UK) and 1% (*v/v*) penicillin/streptomycin in a humidified incubator at 37 °C with 5% CO_2_. Cells (2.5 × 10^6^) were added to a 12 well 3.8 cm^2^ round culture dish for treatment after being washed and resuspended in RPMI without FCS. TNF-α (0.5 ng) was employed as a positive control in a dose-response experiment with increasing concentrations of C3d protein (Merck Chemicals) (0–20 µg/mL). Following 6 h treatment, cells were centrifuged at 500× *g* for 5 min to obtain cell-free supernatants, and a CXCL8 ELISA (Cambridge Bioscience) was performed. A standard curve was created in accordance with suppliers’ instructions and results recorded at 405 nm on a Spectra Max M3 (Molecular Devices, Berkshire, UK).

### 2.8. In Vitro Endothelial Cell Scratch Wound Assays 

Human Umbilical Vein Endothelial Cells (HUVECs) (Sigma) were grown in Endothelial Cell Growth Medium in a humidified incubator at 37 °C with 5% CO_2_, until the required cell density was obtained. For the *in vitro* wound healing assay, 5 × 10^5^ cells were added per well to a 24 well 2 cm^2^ round culture dish for treatment and grown to confluence. To create the wound, a single scratch was created down the center of the well using a sterile pipette tip [33]. Cells were washed with phosphate-buffered saline to remove debris, and cells were incubated in a serum-free medium with and without treatment [C3d (10 or 20 μg/mL); lactoferrin/BPI/MPO (10 μg/mL pool)]. Images of the wound were then taken at 0, 4, or 8 h to assess wound closure with the gap widths quantitatively evaluated using ImageJ software.

### 2.9. Data Analysis 

Results were expressed as mean ± SEM of biological replicates or independent experiments as stated in the figure legends. Data were tested for normality using the D’Agostino and Pearson omnibus normality test. A Student’s *t*-test was used where distribution was normal and when comparison was being made between two groups. The Mann–Whitney U test was employed where data were not normally distributed. Two-way ANOVA was used for independent group comparisons, followed post-hoc by Bonferroni’s multiple comparison test where appropriate. Statistical significance was determined with a *p*-value < 0.05. The data were analyzed with Graphpad Prism 8.0 and SPSS software.

## 3. Results

### 3.1. Increased C3d in Plasma and on Circulating Neutrophils in AATD 

**Figure 1:**
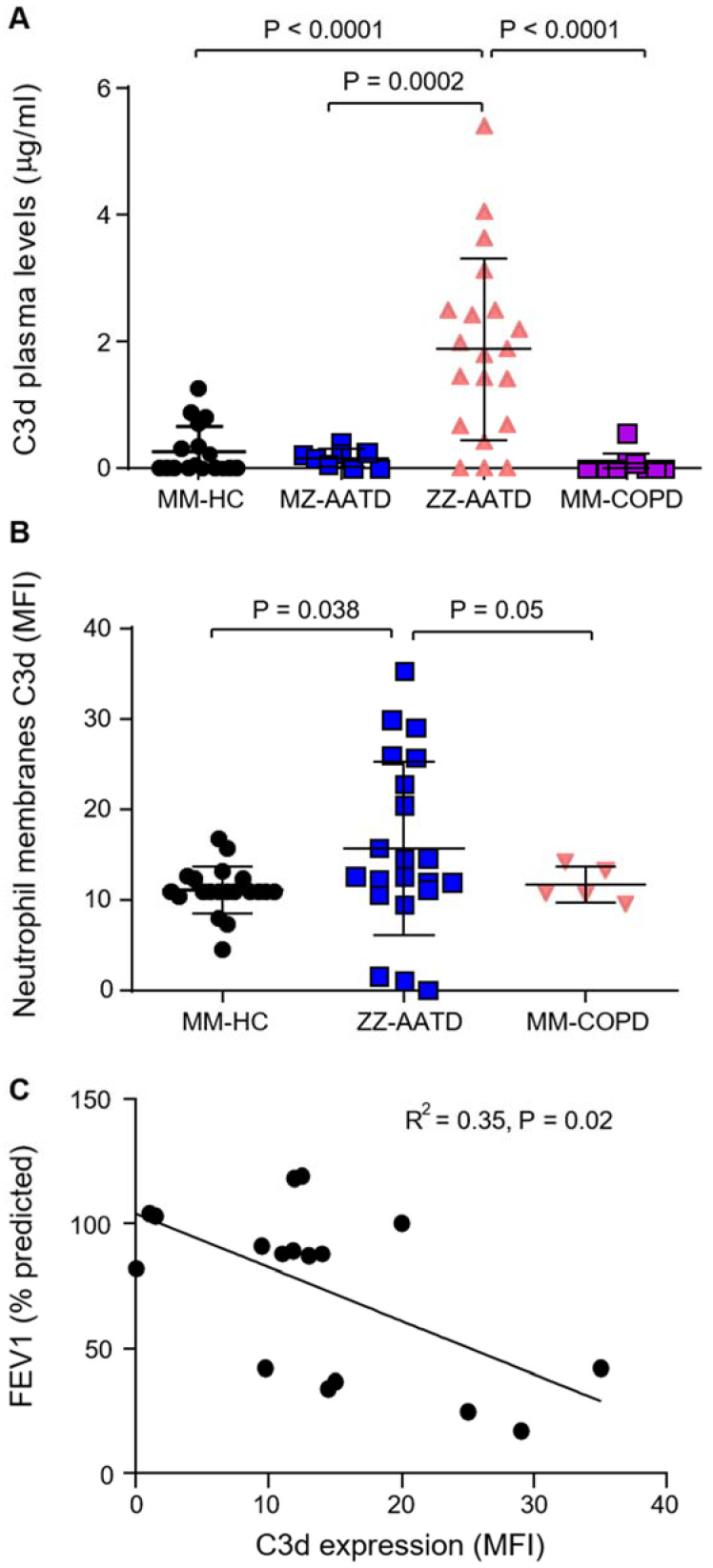
Measurement of C3d in AATD patients. (**A**) Quantification of C3d was performed in the plasma of ZZ-AATD (*n* = 20), MZ-AATD (*n* = 8), FEV1 matched MM-COPD (*n* = 10), and MM-HC individuals (*n* = 17) by ELISA. The mean C3d concentration was increased in ZZ-AATD individuals compared to COPD, MZ individuals, and MM-HC individuals. (**B**) C3d levels were measured on the surface of neutrophil membranes isolated from ZZ-AATD (*n* = 21), FEV1-matched MM-COPD patients (*n* = 5) and MM-HC individuals (*n* = 20) by flow cytometry. Statistical analysis was performed using a Mann–Whitney U test. (**C**) A significant inverse correlation was found between C3d neutrophil membrane expression levels and FEV1 (% predicted) values in ZZ-AATD individuals (R^2^ = 0.35, *p* = 0.02).

To confirm complement activation in AATD, quantification of C3d was performed in plasma of ZZ-AATD (*n* = 20), MZ-AATD (*n* = 8), MM-COPD (*n* = 10), and MM-HC individuals (*n* = 17) by ELISA. The mean C3d concentration was significantly increased in ZZ-AATD (~2 µg/mL) compared to MM-HC (*p* < 0.0001), confirming that complement activation and C3d production are increased in ZZ-AATD individuals (Figure 1A). Moreover, C3d levels were significantly decreased in MZ-AATD (*p* = 0.0002) and AAT-sufficient MM-COPD patients compared to ZZ-AATD (*p* < 0.0001), indicating that the increase in complement activation is related to a lack of AAT, as previously reported [19].

As neutrophilic inflammation is a prominent feature of AATD, ensuing experiments assessed an association between raised plasma C3d levels and circulating neutrophils. By flow cytometry, C3d was found significantly increased on the outer membrane surface of circulating neutrophils of ZZ-AATD compared to MM-HC individuals (*p* = 0.038) (Figure 1B). Moreover, there was no difference in C3d membrane levels between MM-HC and MM-COPD neutrophils (*p* = 0.636), further supporting the concept that the increase in C3d in ZZ-AATD was due to a lack of AAT rather than inflammatory lung disease. Furthermore, FEV1 (% predicted) values were obtained for each patient from the Irish National AATD Registry and correlated with neutrophil membrane-bound C3d (Figure 1C). A significant inverse correlation was found between C3d neutrophil membrane expression levels and FEV1 (% predicted) values in AATD individuals (R^2^ = 0.35, *p* = 0.02). This suggests complement activation as a key player in the pathogenesis of AATD and is in line with the absence of an adequate anti-protease protective screen. Plasma and neutrophils of AATD patients homozygous for the Z-allele were utilized in all subsequent analyses. 

### 3.2. Production of C3d by Neutrophil Membrane-Bound Neutrophil Elastase

**Figure 2:**
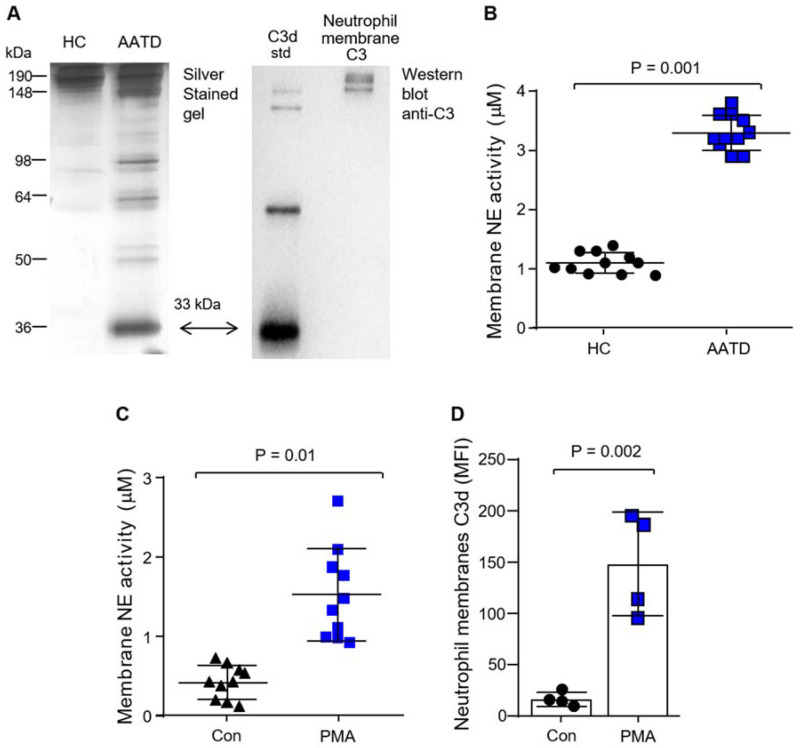
Neutrophil elastase-mediated C3d production. (**A**) Plasma of HC or AATD patients was incubated with 10 μg/mL NE for 1 h at 37 °C. Left panel: silver stain of a 12.5% (*w/v*) non-reducing SDS-PAGE gel demonstrating C3 in HC plasma. The digestion products of C3 proteolysis are observed in AATD plasma. A number of proteolytic bands are seen, including at 33 kDa band corresponding to the C3d fragment. Right hand panel: Western blot of HC neutrophil membrane fractions on a 12.5% (*w/v*) non-reducing SDS-PAGE gel using rabbit polyclonal anti-C3 antibody. A 186 kDa band corresponding to C3 is visible. C3d protein standard (33 kDa) was used as a control. The silver-stained gel and Western blot are representative images of *n* = 3 independent experiments. (**B**) Membrane-bound NE activity was measured on HC and AATD neutrophils by FRET analysis. NE activity levels were significantly higher on the membranes of AATD neutrophils (*p* = 0.001, *n* = 11 biological samples, Student’s *t*-test). (**C**) NE activity was significantly higher on membranes of HC neutrophils following stimulation with PMA for 10 min compared to unstimulated cells (Con) (*p* = 0.01, *n* = 10 biological repeats, Student’s *t*-test). (**D**) C3d expression on HC neutrophils is significantly increased post PMA stimulation compared to unstimulated (Con) neutrophils (*p* = 0.002, *n* = 4 biological repeats, Student’s *t*-test).

AAT is the principal inhibitor of neutrophil-derived proteases, in particular NE. It has previously been shown that C3 can be cleaved by NE to activate complement [34,35]. No difference in the levels of C3 were recorded in AATD patients’ plasma compared to HC samples [19], and thus the aim of this experiment was to determine activation of C3 by NE in the setting of AATD with low plasma levels of AAT protein. For this analysis, NE was added to 1% (*v/v*) HC or AATD plasma, and reactions were incubated for 1 h at 37 °C. Protein samples were then separated under non-reducing SDS-PAGE conditions, and the gels were silver-stained (Figure 2A). Although C3 activation in HC plasma upon addition of 10 µg/mL (338 nM) NE was not apparent, with the addition of NE to AATD plasma, a 33 kDa band corresponding to the correct molecular mass of C3d was visible, indicating that NE may play a role in the increased plasma C3d levels observed in AATD individuals. Moreover, Western blot analysis of non-denaturing SDS-PAGE of HC neutrophil membranes revealed an immuno-band of 186 kDa corresponding to full-length C3 (Figure 2A). This result confirmed the presence of C3 on HC neutrophil membranes. Importantly, localization to the cell membrane could potentially expose C3 susceptible to cleavage by unopposed NE activity. In line with this concept, although free NE activity was not detectable in patient plasma (result not shown), by FRET analysis, NE activity was found significantly increased on the surface of AATD neutrophil membranes compared to HC cells (*p* = 0.001) (Figure 2B). This result corroborates previous work, which demonstrated an increase in the release of primary granules in AATD [13] and raised the possibility that NE located on the surface of neutrophils may cleave C3 leading to C3d production locally, which was next explored.

Neutrophils from HC individuals (5 × 10^6^) were stimulated with PMA (1 µg/mL) at 37 °C for 10 min. Membrane-bound NE was assessed by FRET analysis, with significantly increased levels of active NE detected on neutrophil outer plasma membranes following PMA challenge (*p* = 0.01) (Figure 2C). Moreover, in line with increased NE activity following PMA stimulation, C3d expression on neutrophil plasma membranes was significantly increased compared to unstimulated cells (*p* = 0.002) (Figure 2D). 

**Figure 3:**
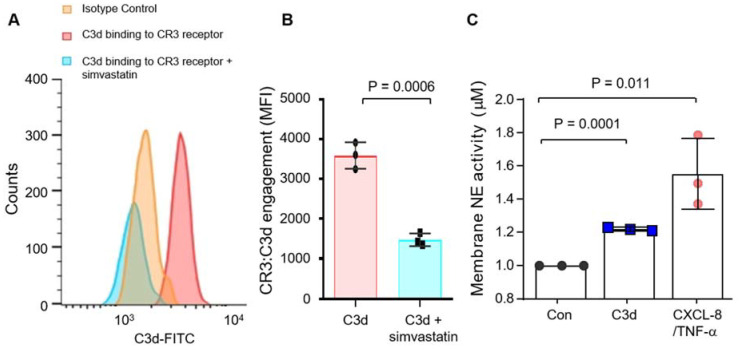
C3d binds neutrophil CR3 and causes the release of neutrophil elastase. (**A**) Flow cytometry analysis of C3d bound to the CR3 membrane receptor on isolated HC neutrophils (red), and in the presence of the CR3 blocker, simvastatin (1 mM). C3d (5 µg) binding was evaluated using C3d mouse monoclonal IgG1. (**B**) The level of CR3/C3d binding was found to be reduced by the inclusion of simvastatin (Student *t*-test). (**C**) NE activity was significantly higher on membranes of C3d or CXCL8/TNF-α challenged neutrophils compared to unstimulated cells (Con) (*p* = 0.0001, *n* = 3 biological repeats, Mann–Whitney U test).

Finally, we investigated the possibility that CR3/C3d interaction may trigger a cycle of inflammation by impacting plasma membrane levels of NE. It has previously been shown that CR3 can bind the C3d fragment of C3 [27]. In the current study, C3d binding to neutrophil CR3 was confirmed by flow cytometry (Figure 3A) and inclusion of simvastatin [27,30], which is known to target CR3 in its ligand-binding domain, blocked C3d receptor engagement (Figure 3A,B). Moreover, HC neutrophils challenged with C3d (40 µg) or a combination of CXCL8/TNF-α, which served as a positive control, demonstrated significantly increased membrane-bound NE activity compared to untreated cells (*p* = 0.011 and *p* = 0.0001, respectively) (Figure 3C). Collectively, these results indicate that C3 is cleaved in AATD by the unopposed activity of NE yielding C3d, and in turn, C3d can increase membrane NE activity levels. This latter result prompted an investigation into the ability of C3d to influence neutrophil degranulation. 

### 3.3. Degranulation of Neutrophil Granule Subtypes in Respone to C3d

**Figure 4:**
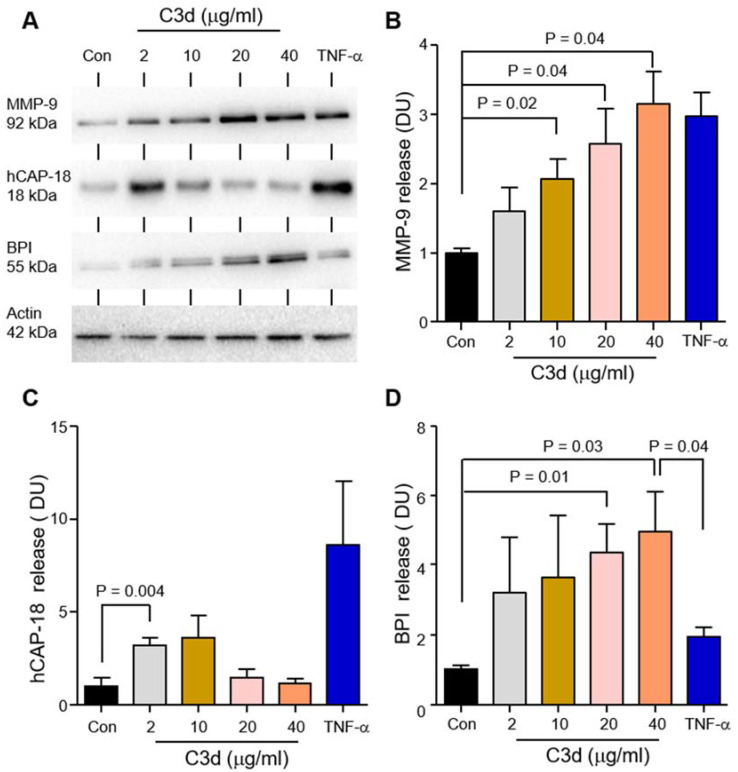
Neutrophil degranulation is increased in response to C3d treatment. Neutrophils isolated from HC individuals were incubated at 37 °C and remained unstimulated (Con) or were stimulated with C3d (2–40 µg/mL) or TNF-α (10 ng / 2 × 10^7^ cells) (**A**–**C**). (**A**) Cell-free supernatants were collected at 10 min and immunoblotted for markers of tertiary granule (MMP-9), secondary granule (hCAP-18), or primary granule (BPI) release. Densitometry of MMP-9 (**B**), hCAP-18 (**C**), and BLPI (**D**) immunobands was performed. Western blot analyses of whole cell lysates demonstrated equal expression levels of β-actin, confirming the use of equal cell numbers per reaction. C3d-challenged neutrophils released significantly greater levels of all three granule types when compared to untreated controls (Con) (*n* = 5 biological repeats, ANOVA followed by Bonferroni post-hoc test for selected groups). All results are expressed as relative densitometry units (DU), with representative Western blots presented.

Data thus far has demonstrated increased C3d levels in AATD plasma and on circulating AATD neutrophils, which prompted the assessment of the effect of C3d on neutrophil function, specifically neutrophil degranulation. Circulating neutrophils (1 × 10^7^) isolated from HC individuals were challenged with increasing concentrations of C3d (0–40 µg/mL) or TNF-α (5 ng/1 × 10^7^) for 10 min at 37 °C. TNF-α was used as a positive control as it has previously been reported to cause secondary and tertiary granule release from neutrophils [31]. Extracellular supernatants were then analyzed for degranulated protein. MMP-9, hCAP-18, and BPI, markers of tertiary, secondary, and primary granule release, respectively, were detected by Western blot analysis (Figure 4A–D). A 2-, 3-, and 3.5-fold increase in the level of MMP-9 was detected after 10 min exposure to 10 (*p* = 0.018), 20 (*p* = 0.037) or 40 µg (*p* = 0.042) C3d (Figure 4A,B). At the higher levels of C3d employed (20 µg or 40 µg), levels of MMP-9 released were comparable to those following TNF-α exposure. Densitometry values of immunoblots for hCAP-18 demonstrated increased degranulation of secondary granules, with 2 µg C3d inducing a significant 3-fold increase in hCAP-18 release (*p* = 0.004) (Figure 4A,C). Of note, a reduction in the level of degranulated hCAP-18 was observed upon increasing the concentration of C3d past 10 µg. hCAP-18 is a precursor molecule for its active form LL-37. We propose that treatment with higher doses of C3d, results in primary granule degranulation, resulting in the release of proteases including proteinase 3, which can cleave hCAP-18 to LL-37, which the Western blot antibody employed in this set of experiments would not detect. To support this concept, a 2-fold increase in the level of BPI, a marker of primary granule release, was detected in extracellular supernatants after 10 min exposure to 20 (*p* = 0.013), or 40 µg C3d (*p* = 0.025) (Figure 4A,D).

**Figure 5:**
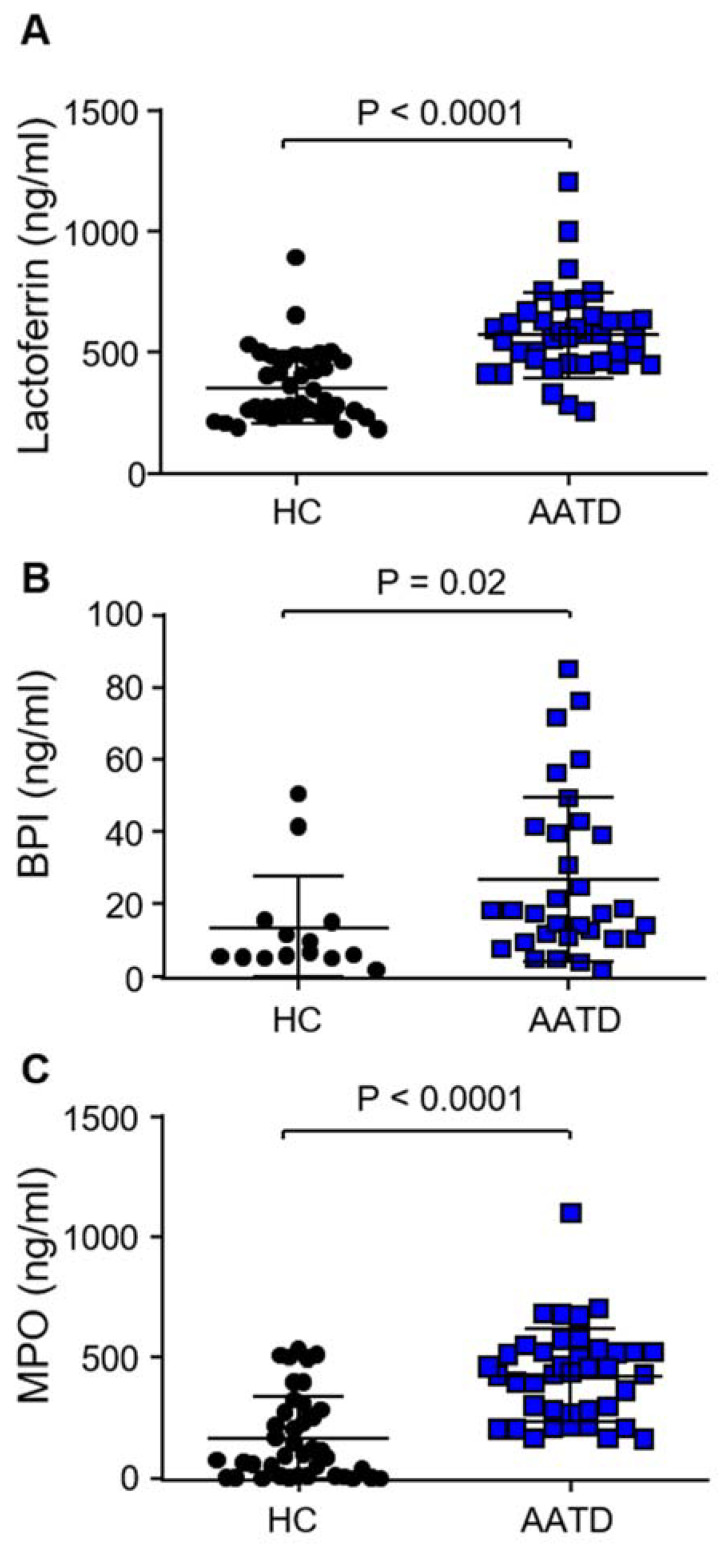
Increased plasma levels of neutrophil degranulated components in AATD. *(***A**) Lactoferrin (*p* < 0.0001, *n* = 40 subjects per group, Mann–Whitney U test), (**B**) BPI (*p* = 0.02, *n* = 14 and *n* = 32 for HC and AATD respectively, Mann–Whitney U test) and (**C**) MPO (*p*<0.0001, *n* = 40 subjects per group, Mann–Whitney U test) quantified by ELISA and expressed as ng/mL, were significantly higher in plasma of AATD individuals compared to HC donors. All measurements are mean ± SEM from biological replicates.

To understand if the increase in degranulation of neutrophil granule components in response to C3d was of importance to patients with AATD, levels of granule components in AATD plasma were measured. The secondary granule protein lactoferrin was previously shown to be increased in the plasma of AATD individuals compared to HC [14], a result further confirmed by the current study (*p* < 0.001) (Figure 5A). This set of experiments was expanded to include ELISA measurements of the primary granule component BPI. BPI levels were significantly increased in AATD plasma compared to HC samples (*p* = 0.02) (Figure 5B). Finally, recordings of plasma levels of MPO, a further primary granule marker, demonstrated significantly increased levels in AATD plasma compared to HC (*p* < 0.0001) (Figure 5C). Collectively, these results indicate increased primary granule release by AATD neutrophils in vivo and suggest that increased C3d may contribute to this observed event. 

### 3.4. Secretion of Pro-Inflammatory CXCL8 in Response to C3d

**Figure 6:**
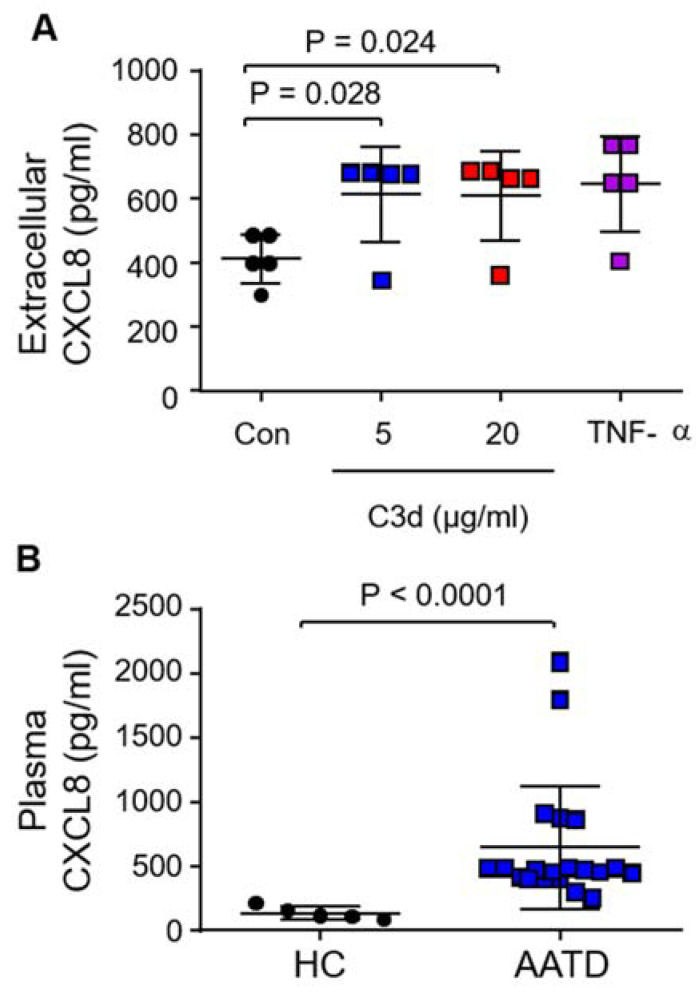
C3d induced secretion of pro-inflammatory CXCL8. (**A**) Extracellular supernatants of C3d-challenged HL-60 cells were analyzed by ELISA for the presence of CXCL8. C3d (5 µg or 20 µg/mL) treatment released significantly greater levels of CXCL8 when compared to control (Con) untreated cells (*p* = 0.03, *n* = 5 independent experiments, ANOVA followed by Bonferroni post-hoc test for selected groups). (**B**) Levels of soluble CXCL8 were significantly higher in AATD plasma samples (*n* = 25) when compared to healthy controls (*n* = 7; HC) (*p* < 0.0001, Student’s *t*-test). Measurements are mean ± SEM.

Neutrophils possess the ability to produce CXCL8, a potent endogenous chemoattractant in a dose- and time-dependent manner in response to stimulants such as TNF-α [36], and thus ensuing experiments examined the release of CXCL8 in response to C3d. For this, HL-60 cells, a neutrophil-like cell line was utilized to preclude the possibility of false-positive results by co-purified immune cells known to produce high levels of CXCL8 [37]. The mean C3d concentration previously recorded in AATD airway and plasma samples was 34.6 µg/mL and 2 µg/mL, respectively [19]. For the current set of experiments, we employed physiological levels of 5 or 20 μg/mL C3d. HL-60 cells were treated with C3d (0, 5, 20 µg) or TNF-α (0.5 ng/2.5 × 10^6^) as a positive control for CXCL8 release. Following 6 h treatment, extracellular culture supernatants were collected and analyzed for CXCL8 by ELISA. HL-60 cells released significantly more CXCL8 in response to 5 µg (612.6 ± 66.5 pg/mL, *p* = 0.028) and 20 µg C3d (610 ± 62 pg/mL, *p* = 0.024) compared to untreated cells (413.4 ± 35 pg/mL) (Figure 6A). In corroboration of this result, *ex vivo* examination of CXCL8 plasma levels demonstrated a substantial increase in circulating CXCL8 in AATD compared to HC samples (*p* < 0.0001) (Figure 6B). These results indicate that the neutrophil C3d:CR3 signaling axes may potentially influence CXCL8 levels in the circulation of AATD patients.

### 3.5. The Impact of C3d on Wound Healing of Vascular Endothelial Cells

**Figure 7:**
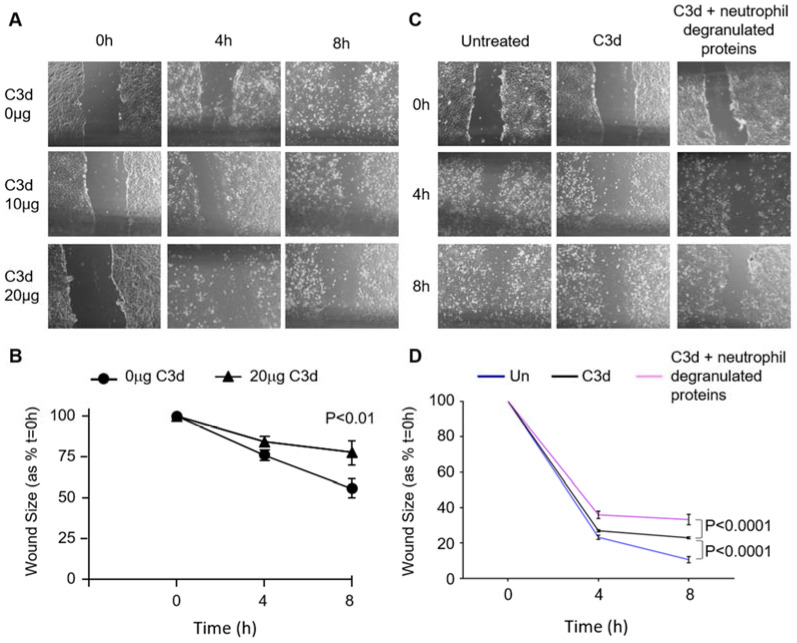
The effect of C3d on endothelial cell migration. (**A**) Representative images of *in vitro* wound healing assays of HUVECs either untreated or treated with C3d (10 or 20 µg/mL). Microphotographs of the wounded area were taken immediately after the scratch (0 h) and 4 h or 8 h. (**B**) Wound healing rates were significantly decreased by 20 μg/mL C3d (*p* < 0.0001) (*n* = 3 independent experiments). (**C**) HUVECs remained untreated or were treated with 20 μg/mL C3d ± neutrophil degranulated proteins (10 μg/mL MPO/lactoferrin/BPI pool). (**D**) Wound healing rates were significantly decreased in C3d (*p* < 0.0001) and C3d + degranulated proteins at 8 h (*p* < 0.0001) (*n* = 3 independent experiments, ANOVA). Images in panels (**A**,**C**) were taken at 20× magnification.

The Z allele of SERPINA1 is a known risk factor for the development of systemic ANCA-vasculitis [38]. As endothelial cells play a crucial role in small vessel vasculitis and the vascular response to inflammation, ensuing experiments assessed the effect of increased C3d on local endothelial cell processes. An *in vitro* wound-healing assay was used to examine the effect of C3d on endothelial cell migration, a key process in wound healing and angiogenesis. HUVEC cells were grown to confluence in 24-well plates, and a single scratch was created down the center of the well to produce a wound. Then, cells were incubated with or without C3d treatment, with images recorded at 0, 4, and 8 h to assess wound closure. For the current set of experiments, we employed 10 or 20 μg/mL C3d, which was within the physiological range. HUVEC untreated controls showed more evidence of cell scatter-driven migration compared to cells in the presence of C3d (20 µg) after 8 h incubation (*p* < 0.01) (Figure 7A,B). Moreover, exposure of HUVECs to C3d in combination with neutrophil granule proteins MPO, BPI, and lactoferrin (10 µg pool) significantly decreased cell migration compared to untreated cells (*p* < 0.0001), or those treated with C3d (20 µg) only (*p* < 0.0001) (Figure 7C,D). Collectively, these results demonstrate the negative impact of C3d, and more so, C3d in combination with neutrophil primary granule components on endothelial cell migration, with the potential consequence for wound repair and AATD-related vasculitis.

## 4. Discussion

Neutrophilic inflammation is a prominent feature of both COPD and AATD. However, neutrophils in AATD are a clinically important cell to study as patients have a significantly higher burden of neutrophils in their airways [39,40] and an elevated neutrophil chemotactic index in bronchoalveloar lavage fluid [41]. C3d is considered a robust marker of complement activation [42], and in the current study, the mean C3d level in plasma was significantly increased in AATD compared to MZ-AATD individuals or AAT-sufficient COPD patients. This result indicates that complement activation may be occurring as a result of low levels of circulating AAT rather than solely due to inflammation. Exploring the mechanisms underlying dysregulated complement activation demonstrated that increased NE activity localized to the outer plasma membrane of neutrophils can cleave C3 resulting in the production of C3d. Collectively, these results demonstrate that C3d is a product of the NE-driven inflammatory cycle in AATD, and in association with CR3, functions to trigger increased neutrophil degranulation and pro-inflammatory leucocyte CXCL8 secretion. 

Dysregulated neutrophil degranulation is an important feature in the pathogenesis of AATD. It has previously been demonstrated that AAT can modulate TNF-α induced degranulation processes in a dose-dependent manner, with increased plasma levels of secondary and tertiary neutrophil granule components detected in AATD [14]. Moreover, primary granules of neutrophils contain an arsenal of pro-inflammatory and anti-microbial proteins, including serine proteases NE, proteinase 3 (PR3), and cathepsin G. Indeed, once degranulated, NE can rebind membranes and exosomes of neutrophils [43]. In line with this concept, the neutrophil membrane proteome in AATD-COPD was shown to differ significantly from that in AAT sufficient-COPD as demonstrated by increased membrane abundance of primary granule proteins, including NE. The signaling mechanism underlying increased degranulation included increased Rac2 activation, resulting in proteinase-activated receptor 2 activation by serine proteinases and enhanced reactive oxygen species production [44]. In the current study, we examined the potential pro-inflammatory role of C3d on neutrophil degranulation. Following treatment of HC neutrophils with increasing concentrations of C3d, enhanced release of tertiary (MMP9), secondary (HCAP-18), and primary granule components (BPI, NE) were recorded. Of note, results demonstrating the ability of membrane-bound NE to generate C3d, and, in turn, the ability of C3d to trigger granule release yielding further localized membrane NE activity could represent a self-perpetuating cycle of C3d production and inflammation. However, to support this proposal, further experiments involving specific C3d blockade in *in vitro* and in vivo AATD models would help determine whether C3d elevation is a driver or an effect. Moreover, these results demonstrate that the *in vitro* challenge of neutrophils with C3d is a potent stimulus resulting in the release of all three granule types, which is in contrast to TNF-α, which is effective at secondary and tertiary release only. Additionally, further experiments confirming the ability of C3d to cause CXCL8 release could compare the impact to C3a, an additional inflammatory marker related to complement activation [19]. C3a is a direct product of C3 cleavage, determined by C3a-desarg levels. When analyzed with respect to the presence of airway obstruction, a significant difference was observed in plasma levels of C3a in ZZ-AATD individuals compared to HC samples [19].

The recorded induction of granule release by C3d may play a role in the pathogenesis of AATD, including degradation of tissue by excessive release of proteases or aiding in the development of autoantibodies. For example, the tertiary granule protein MMP-9 can degrade major extracellular matrix structures, including laminin and collagen [45], and is implicated in lung function decline [46]. NE is involved in destructive lung tissue damage and modulates inflammation by cleaving cell surface receptors and adhesion molecules [47]. Although PR3 is reported to have less elastolytic ability than NE [48], anti-PR3 autoantibodies have nonetheless been proposed to cause significant vascular and endothelial injury (147). In support of the concept of autoimmunity in AATD, small studies have suggested that the presence of the Z-allele is associated with the development of anti-neutrophil cytoplasmic antibodies (ANCA) [23,49], causing aberrant neutrophil activation and ANCA-associated vasculitis [38]. Our own studies have previously shown AATD as a risk factor for developing neutrophilic ulcerative panniculitis [50], increased anti-lactoferrin autoantibodies [14], and increased titers of anti-CCP in MZ-AATD patients with rheumatoid arthritis (RA) [51]. In patients with cystic fibrosis (CF), increased anti-BPI autoantibodies have been detected [52,53], but although data of the current study demonstrate that BPI levels are significantly increased in the plasma of AATD individuals, no link between AATD and anti-BPI antibodies has been established [54]. 

Complement activation has been reported to play a role in ANCA-associated vasculitis, with circulating levels of complement activation fragments increased in the plasma of those with active disease [55]. Of relevance, ANCA-associated vasculitis, in particular, GPA, is a reported manifestation of AATD. Avacopan, an orally-administered inhibitor of the complement fragment 5a (C5a) receptor, demonstrated encouraging results for the treatment of ANCA-associated vasculitis [56]. Moreover, current treatment for AATD patients includes AAT augmentation therapy, which involves weekly infusions of plasma purified AAT. Intravenous AAT augmentation therapy slows emphysema progression by decreasing loss of lung density [57]. More recently, however, AAT therapy was demonstrated to reduce both circulating and airway levels of C3d in individuals with AATD [19]. Moreover, evidence exists to support the use of AAT augmentation therapy in the treatment of GPA vasculitis [25] and to effect clinical remission of panniculitis following single-dose AAT therapy [50]. To contribute to the expansion of knowledge in this field, we explored the impact of increased complement activation and neutrophil degranulation on endothelial cell wound repair. It must be noted that a limitation of the current study is the lack of an in vivo model to confirm the impact of AAT therapy on complement activation and wound repair. Moreover, the inhomogeneity between the number of AATD patients recruited to the study and control groups is a further limitation. Nevertheless, our *in vitro* data demonstrate that endothelial cell migration was significantly reduced in the presence of C3d and neutrophil granule proteins. Collectively, these data indicate that targeting CR3/C3d signaling in neutrophils, thereby modulating the C3d cycle of inflammation, may represent a new therapeutic avenue in AATD-related disease.

## 5. Conclusions

Increased complement activation and C3d levels have been demonstrated to represent key processes in the pathogenesis of AATD. Greater C3d concentrations retain the ability to bind CR3 on the neutrophil membrane resulting in increased release of primary, secondary, and tertiary granule components. C3d also induces the release of CXCL8, a key neutrophil chemoattractant, potentially amplifying the neutrophilic inflammatory burden. Moreover, within the blood vasculature, the presence of C3d and granule proteins may slow endothelial cell migration and wound repair. As AAT augmentation therapy impacts C3d production [19], this study supports future research into the therapeutic use of AAT augmentation therapy in conditions other than AATD in which C3d has been implicated, including systemic lupus erythematosus, ANCA-associated vasculitis, and RA.

## Figures and Tables

**Table 1:** Characteristics of healthy controls, ZZ-AATD and MM-COPD patients.

	ZZ-AATD	MZ-AATD	MM-COPD	HC
Number of subjects	80	8	10	40
Age, years (mean ± SD)	49.62 ± 13.92	55.86 ± 15.08	70.86 ± 6.71	30.38 ± 5.01
FEV1 (% predicted)	75.54 ± 33.79	70.86 ± 24.48	57.3 ± 23.32	99.56 ± 7.9
FEV1/FVC (% predicted)	59.15 ± 20.72	61.86 ± 17.28	50 ± 16.94	80.15 ± 6.52
DLCO (% predicted)	64.58 ± 24.98	81.00 ±21.43	36 ± 10.86	-

Definition of abbreviations: SD = standard deviation, FEV1 = forced expiratory volume in one second, DLCO = diffusion capacity for carbon monoxide.

## Data Availability

Data available upon request.
